# Seasonal effect of milk yield and blood metabolites in relation to ketosis of dairy cows fed under a high ambient temperature

**DOI:** 10.14202/vetworld.2021.2392-2396

**Published:** 2021-09-16

**Authors:** Sumpun Thammacharoen, Sapon Semsirmboon, Somchai Chanpongsang, Narongsak Chaiyabutr, Pawares Panyasomboonying, Paweenut Khundamrongkul, Peeravit Puchongmart, Worapruch Wichachai

**Affiliations:** 1 Department of Physiology, Faculty of Veterinary Science, Chulalongkorn University, Pathumwan, Bangkok 10330, Thailand; 2 Department of Animal Husbandry, Faculty of Veterinary Science, Chulalongkorn University, Pathumwan, Bangkok 10330, Thailand; 3 The Academy of Science, The Royal Society of Thailand, Dusit, Bangkok 10300, Thailand; 4 Queen Saovabha Memorial Institute, The Thai Red Cross Society, Bangkok 10330, Thailand.

**Keywords:** dairy cattle, environmental temperature, heat stress, lactation, subclinical ketosis

## Abstract

**Background and Aim:**

Metabolism and environment are closely related. Under high ambient temperature (HTa), dairy cows may have different energy metabolism during summer and winter. The present study was carried out to investigate the effect of HTa on the milk yield and blood concentration of beta-hydroxybutyrate (BHBA) and glucose at the herd level.

**Materials and Methods:**

One large dairy farm in Thailand with more than 100 crossbred Holstein cows milked each month was selected. The first experiment was performed on non-lactating cows to determine the normal daily concentrations of blood BHBA and glucose. Under the HTa condition, there was no significant change in blood BHBA and glucose concentrations. The second experiment was performed using a prospective cohort clinical design to demonstrate the seasonal effect on milk yield and blood BHBA as an indication of energy metabolism at the herd level.

**Results:**

The temperature and humidity index for the winter (78.1±0.5) and summer (83.4±0.7) periods differ significantly. The average milk yield during the winter period was 17.8% higher than during the summer period. The reduction of body condition score (BCS) during early lactation was significant in the winter cows. Both higher milk yield and lower BCS in the winter cows suggested a state of negative energy balance. However, there was no difference in blood BHBA and glucose concentrations between winter and summer cows. The effect of HTa on insulin signaling appeared to be a counterbalancing factor for the ketogenic status. Based on the present results, it would be interesting to further investigate the incidence of subclinical and clinical ketosis in a dairy farm under tropical conditions.

**Conclusion:**

The present experiment revealed that HTa during summer decreased milk yield in dairy cows fed under tropical conditions. Higher milk yield in winter caused a greater reduction of BCS and suggested a greater negative energy balance. However, there was no seasonal effect on blood BHBA and glucose concentrations.

## Introduction

The main climate of Thailand is tropical savannah based on the Köppen–Geiger climate classification [1]. There are generally three main seasons in Thailand [2]: Rainy season (mid-May to mid-October), winter season (mid-October to mid-February), and summer season (mid-February to mid-May). Using herd level analysis, we had previously demonstrated that milk yield per day from dairy cows during summer was 16% lower than during winter [3]. The negative effect of summer months on mammary gland function inspired us to investigate the seasonal effect on the metabolic stress during periparturition and the early phase of lactation.

It is well known that dairy cows are challenged by a shortage of energy supplied to the mammary gland during the early phase of lactation. This phenomenon comes in part from the lower rate of gradually increased feed intake (FI) and the higher rate of milk synthesis during this period [4]. Due to this negative energy balance, available fat from adipose tissue is the main source of energy metabolism. The transition of carbohydrate to fat metabolism could be a factor for an increase in ketones in the body (hyperketonemia) and for clinical disease or impaired milk production [5]. In dairy cows, an increase in fat metabolism and the whole-body adaptation to this condition can be monitored by the blood beta-hydroxybutyrate (BHBA) concentration. BHBA is the predominant ketone in the body. The incidence of hyperketonemia during early lactation (1-16 days in milk [DIM]) has been investigated at herd level. The peak incidences for both subclinical and clinical ketosis were 22% and 3%, respectively, during 5-7 DIM [6]. In addition, hyperketonemia during early lactation has been shown to cause an increase in health risk and a decrease in milk production [7]. Hyperketonemia development was also more sensitive in dairy cows during prepartum with higher body condition scores (BCSs) [8]. Insulin signaling apparently intervened in the relationship between BCS and the sensitivity of hyperketonemia development. Glucose-activated insulin secretion and insulin-activated glucose uptake were blunted in hyperketonemic cows [9]. In addition, the daily pattern of BHBA concentrations was different between dairy cows fed with high- or low-energy diets [10]. These studies suggest that all factors related to energy metabolism during peripartum determine the outcome of the adaptation to this metabolic stress. Under the high ambient temperature (HTa) condition, we recently demonstrated that HTa decreased milk yield by direct (to the mammary gland) and indirect (decrease in FI) mechanisms [11]. In short-term heat exposure, the activation of insulin release (both basal and stimulated) could increase glucose utilization and decrease adipose tissue lipid mobilization [12].

We hypothesize that the postpartum concentration of BHBA from lactating cows during summer is different from that during winter under the HTa condition.This study aimed to investigate the influence of HTa on the energy metabolism in dairy cows. The knowledge is the part of livestock welfare that needs an appropriate mean to support a good life quality of dairy cows fed under tropical regions.

## Materials and Methods

### Ethical approval

This experiment followed the guidelines of the Ethical Principles and Guidelines for the Use of Animals for Scientific Purposes by the National Research Council of Thailand and was approved by the Institutional Animal Care and Use Committee (IACUC) in accordance with the university regulations and policies governing the care and use of experimental animals (animal use protocol no. IACUC1831100).

### Study period and location

The data were collected on a large commercial dairy farm in Ratchaburi Province, Thailand (latitude 13.823360°, longitude 99.878351°) from January 2019 to January 2020. The average temperature, humidity and total rainfall of the study area were 27.6 ± 2.1ºC, 70.6±4.0% and 1,012 mm, respectively. This farm contained more than 100 crossbred Holstein cows (>87.5% Holstein) milked each month. The samples were processed at the Small Ruminant Physiology and Nutrition Research Laboratory, Faculty of Veterinary Science, Chulalongkorn University.

### Experiment 1: Daily pattern of glucose and BHBA in dry cows fed under HTa

The first experiment was performed to evaluate the daily fluctuation of blood glucose and BHBA, an important measure for the cross-sectional study in the second experiment under the HTa condition. Five non-lactating dairy cows were selected for studies on their blood BHBA and glucose concentrations. The cows were housed within single large pens (20×5 m) with five tie stalls. Food as a total mixed ration was provided twice per day, and water was provided as often as desired. Regular management to alleviate heat stress at this farm using misty fan cooling was applied to all cows from 13.00 to 14.00. For each cow, the blood sample was collected from the ear vein using a 21 gauge needle every 2 h for 24 h (08.00-06.00, +1). The concentrations of BHBA and glucose were immediately measured usingBHBA and glucose meters (BHBCheck Plus; PortaCheck Inc., Moorestown, NJ, USA). The measurements were based on the chemical enzymatic reaction specific for either BHBA or glucose in milk, with validation based on Sailer *et al*. [13]. After blood collection, the rectal temperature (Tr) was measured from each cow using a digital thermometer. The ambient conditions, including ambient temperature (Ta) and relative humidity (RH), were recorded on each sampling day using a wet and dry bulb thermometer. RH was calculated based on dry and wet bulb temperature differences using a psychrometric chart. The temperature and humidity index (THI) was determined according to Saipin *et al*. [11] as follows:

THI=((1.8×Ta)+32)−[(0.55-(0.0055×RH))×((1.8×Ta)-26.8)].

### Experiment 2: The seasonal effect on milk yield and blood BHBA and glucose concentrations

A cross-sectional study was performed, and a prospective cohort design was used to compare the effect of season on BCS, the average daily milk yield, and the concentrations of blood BHBA and glucose. Cows from this herd were eligible to be enrolled if they were calved within the period of summer (April-June; n=25) and winter (October-December; n=25). The BCS from each eligible cow was evaluated prepartum based on a 5-score system [8]. Evaluation of BCS was performed independently by three trained veterinary students, and the scores were averaged. Within the 2^nd^ week postpartum (day 7-14 postpartum), BCS, respiratory rate (RR), and blood BHBA and glucose were evaluated for each cow. All procedures were done during 11.00 -13.00, according to our first experiment. The daily milk yield from each cow during early lactation (6-week postpartum) was averaged from the weekly recorded milk yield. The ambient conditions (Ta, RH, and THI) were recorded and calculated for each sampling day as described in the first experiment.

### Statistical analysis

All data are presented as the mean and standard error of measurement. Data that contain time as a factor were analyzed using repeated one-way analysis of variance (ANOVA). Data that contained two factors were analyzed using repeated two-way ANOVA. Data from two seasons were compared using an unpaired t-test. The significance was declared at p<0.05.

## Results

### Experiment 1: Daily pattern of BHBA and glucose under HTa condition

**Figure-1:**
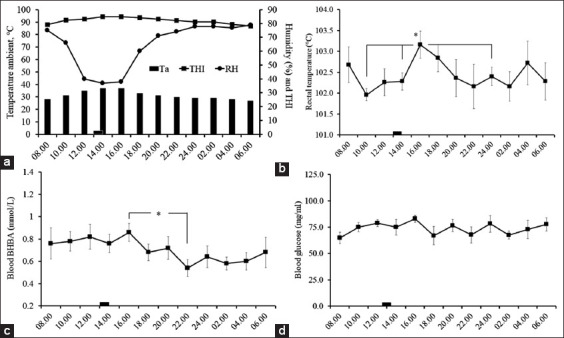
The ambient conditions on the experimental day every 2 h from 800 to 600 <+1>. The bar at the horizontal scale between 1300 and 1400 represents the time of misty fan cooling (a). The rectal temperature (b), blood beta-hydroxybutyrate (c), and glucose (d). *The significant difference by Student’s *t*-test between highest and lowest values (p<0.05).

The average Ta, RH, and THI were 31±1.0°C, 65±4.4%, and 82±0.7, respectively. The range of Ta on the day of experiment was 10°C (Figure-1a). Although the overall Tr values throughout the day and night (Figure-1b) were not significantly different (F_11,44_=1.69, p>0.05), comparisons between the highest Tr point (103.2±0.32°C, 16.00) to many lower Tr points at night and early morning (10.00, 14.00, and 02.00) were significantly different (T_4_=2.77, 3.07, and 3.30, p<0.05). There were no significantly different values of blood BHBA (Figure-1c) and glucose (Figure-1d) throughout the 24 h period (F_11,44_=1.14 and 0.72, p>0.05). However, the comparison between the highest (0.86±0.08 mmol/L, 16.00) and lowest (0.54±0.07 mmol/L, 22.00) blood BHBA was significantly different (T_4_=2.87, p<0.05).

### Experiment 2: The effect of season on milk yield and blood BHBA and glucose concentrations

The average summer Ta and THI (35.6±0.6°C and 83.4±0.7, respectively) were significantly higher than winter Ta (31.1±0.4°C and 78.1±0.5, respectively, T_48_=6.15 and 5.73, p<0.05). The RH in summer (39.4±1.4%) and winter (40.2±2.0%) was not significantly different (T_48_=0.36, p>0.05).

**Figure-2:**
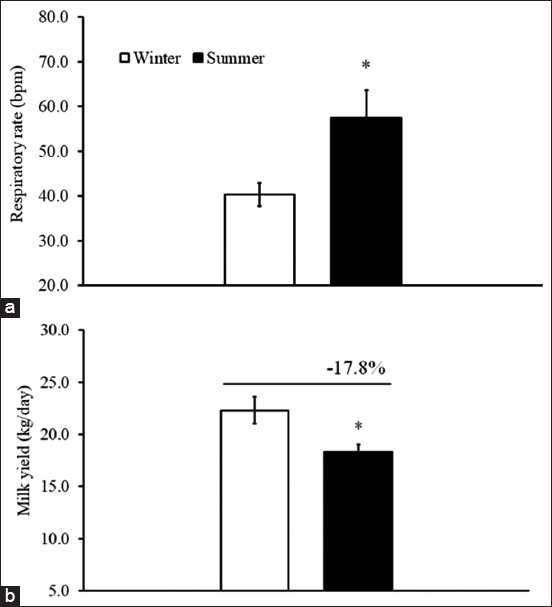
The average respiratory rate (breaths per min, bpm) from winter (open bar) and summer (dark bar) cows (a) and the average daily milk yield during early lactation (b). *The significant difference by Student’s t-test between winter and summer cows.

**Figure-3:**
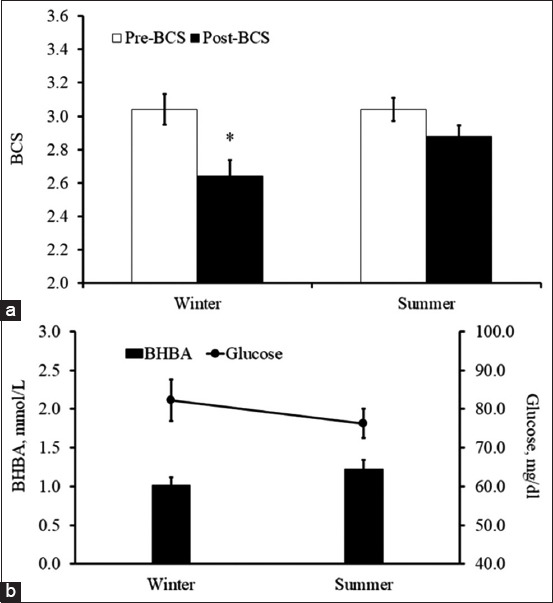
The average body condition score during prepartum (pre-body condition score [BCS], open bar) and during postpartum (post-BCS, dark bar) from winter and summer cows (a). Blood concentration of beta-hydroxybutyrate (black bar, right scale) and glucose (line, left scale) from winter and summer cows (b). *The significant difference by Student’s t-test between winter and summer cows.

The average RR during the summer months was significantly higher than during the winter months (Figure-2a, T_29_=2.47, p<0.05). The average milk yield during the summer months was significantly lower (17.8%) than during the winter months (Figure-2b, T_29_=2.69, p<0.05). Likewise, an effect from the season on BCS was seen (Figure-3a, F_1,48_=13.36, p<0.05). This effect mainly came from a significant reduction in BCS during winter (T_48_=3.43, p<0.05). There were no differences between theconcentrations of blood BHBA and glucose from the summer and winter months (Figure-3b, T_29_=1.41 and 0.90, p>0.05).

## Discussion

The data revealed the effect of HTa on milk yield from dairy cows fed under tropical conditions. Under these conditions, milk yield from the winter months was 17.8% higher than from the summer months. This effect corresponded to a reduction in BCS during winter. Interestingly, the concentrations of blood BHBA and glucose were not affected by the season.

The climatic condition of the present experiment is typically hot and humid. Similar to our previous report [3,14], our findings emphasize that livestock fed in a tropical area is under some degree of heat stress in both the winter and summer periods. In addition, the present experiment showed the comparable effect of HTa on milk yield that was 17.8% lower in the summer months than in the winter months [3]. This result suggests that summer cows were under more stress than winter cows. Both direct and indirect effects of HTa on milk synthesis have been in part demonstrated in dairy cows and goats [11,15]. In addition, the physiological responses to heat stress may influence the biochemical mechanisms related to energy metabolism.

It is well known that during early lactation, dairy cows are at a stage of energy shortage and are at risk of hyperketonemia due to the transition of carbohydrate to lipid metabolism [5]. The whole-body adaptation for the initiation of hyperketonemia depends in part on the sensitivity of insulin signaling. It has been demonstrated that high prepartum BCS dairy cows develop hyperketonemia when compared with low or medium BCS dairy cows [8]. Obesity or high body mass index is a well-known condition where the sensitivity of insulin signaling is reduced in laboratory rodents [16]. At herd level, we demonstrated that prepartum dairy cows had a similar BCS in both seasons. However, dairy cows in winter months had lower BCS during early lactation. The result apparently came in part from the higher milk yield during winter and suggests that winter cows have a greater negative energy balance than summer cows. However, there was no difference in blood BHBA and glucose between winter and summer cows. The reason for the latter result could probably be explained in part by the influence of HTa on the sensitivity of insulin signaling. Under conditions of HTa, insulin signaling is apparently more sensitive than in normal ambient temperatures [12]. Although the ambient conditions during winter in the present experiment were different from the conditions during summer, we considered as well that the Ta and THI (31.1±0.4°C and 78.1±0.5, respectively) in the winter months were higher than the normal threshold of comfortable conditions. Insulin sensitivity evaluation is the crucial information for this hypothesis. Unfortunately, insulin signaling was not determined in the present clinical experiment. This was due in part to the limitation of the test. Insulin sensitivity could not be changed, even by feeding dairy cows with a high-energy diet [17]. Studying insulin signaling has limitations because the test itself is mainly influenced by the physiological stage of gestation and lactation [18]. In fact, under the short-term HTa exposure experiment, both basal and activation of insulin release were comparable with the normal Ta control condition [12]. Instead of a simple glucose tolerance test, the hyperinsulinemic-euglycemic clamp test should be selected for insulin signaling measurement to test the hypothesis of whether insulin signaling is the counterbalancing mechanism for the HTa effect on blood BHBA.

## Conclusion

The present experiment revealed the effect of HTa on milk yield and blood BHBA at herd level during the winter and summer periods. The average daily milk yield from winter cows was higher than from summer cows. This may have contributed to the lower BCS in winter cows and suggests that winter cows experienced a greater negative energy balance. However, winter and summer concentrations of blood BHBA were not significantly different. The latter result may probably be explained in part by the effect of HTa on insulin signaling. The risk of developing hyperketonemia for dairy cows fed under HTa during winter and summer in a tropical climate may depend in part on environmental and physiological adaptations.

## Authors’ Contributions

ST, SS, and NC: Contributed to the conception and designed the study. ST: Contributed reagents/materials/analysis tools. SS, ST, SC, PPa, PK, PPu, and WW: Performed animal experiments. ST: Analyzed the data and performed the statistics. ST: Wrote and revised the paper. All authors contributed to the final version of the manuscript and then read and approved the final manuscript.
